# Structure and antimicrobial activity of NCR169, a nodule-specific cysteine-rich peptide of *Medicago truncatula*

**DOI:** 10.1038/s41598-021-89485-w

**Published:** 2021-05-10

**Authors:** Noriyoshi Isozumi, Yuya Masubuchi, Tomohiro Imamura, Masashi Mori, Hironori Koga, Shinya Ohki

**Affiliations:** 1 Center for Nano Materials and Technology (CNMT), Japan Advanced Institute of Science and Technology (JAIST), 1-1 Asahidai, Nomi, Ishikawa 923-1292 Japan; 2 Ishikawa Prefectural University, 1-308, Suematsu, Nonoichi, Ishikawa 921-8836 Japan

**Keywords:** Symbiosis, Solution-state NMR

## Abstract

A model legume, *Medicago truncatula*, has over 600 nodule-specific cysteine-rich (NCR) peptides required for symbiosis with rhizobia. Among them, NCR169, an essential factor for establishing symbiosis, has four cysteine residues that are indispensable for its function. However, knowledge of NCR169 structure and mechanism of action is still lacking. In this study, we solved two NMR structures of NCR169 caused by different disulfide linkage patterns. We show that both structures have a consensus C-terminal β-sheet attached to an extended N-terminal region with dissimilar features; one moves widely, whereas the other is relatively stapled. We further revealed that the disulfide bonds of NCR169 contribute to its structural stability and solubility. Regarding the function, one of the NCR169 oxidized forms could bind to negatively charged bacterial phospholipids. Furthermore, the positively charged lysine-rich region of NCR169 may be responsible for its antimicrobial activity against *Escherichia coli* and *Sinorhizobium meliloti*. This active region was disordered even in the phospholipid bound state, suggesting that the disordered conformation of this region is key to its function. Morphological observations suggested the mechanism of action of NCR169 on bacteria. The present study on NCR169 provides new insights into the structure and function of NCR peptides.

## Introduction

Root nodules of legume plants are formed by symbiosis with rhizobia, which are nitrogen-fixing bacteria in the soil. This symbiotic organ can produce ammonia as a nutrient source directly from nitrogen in the atmosphere^1^. In the nodule cells, rhizobia are covered with a plant-derived symbiosome membrane and differentiate into the symbiotic form called bacteroid. Inverted repeat-lacking clade (IRLC) legumes are known to have unique bacteroid differentiation mediated by nodule-specific cysteine-rich (NCR) peptides^2^.

*Medicago truncatula*, an IRLC legume, is a well-known model legume that establishes symbioses with its symbiotic partner *Sinorhizobium meliloti*^3^. *M. truncatula* has over 700 *NCR* genes, of which 639 NCR peptides have been detected^4,5^. They are specifically expressed in the nodules as secretory peptides and act on symbiosomes to induce proper bacteroid differentiation^6,7^. Because *M. truncatula* has a wide variety of NCR peptides compared to other IRLC legumes, their functions are thought to be redundant^4^. Despite this hypothesis, two NCR peptides, NCR169 and NCR211, are indispensable factors for nitrogen-fixing symbiosis^8,9^. The gene expression profiles of the deficient mutants corresponding to *ncr169* and *ncr211* are very similar, but the induced bacteroid morphologies are different^10,11^. Experiments using plants showed that the mutant *Δncr169* is unable to form differentiated bacteroids^8^, whereas the mutant *Δncr211* is capable of bacteroid differentiation, but the bacteroids cannot survive^9^. Therefore, these two essential factors likely have their individual roles in bacteroid development and survival.

NCR peptides are classified as defensin-like families because of their conserved cysteine residues. In general, highly cationic NCR peptides are expected to have an antimicrobial activity similar to that of defensins. In fact, NCR247 and NCR335 have antimicrobial activity against various bacteria and fungi^12,13^. Furthermore, an extensive in vitro study using 19 NCR peptides revealed that NCR peptides with high isoelectric points (pI > 9) have antimicrobial activity against *Candida albicans*^14^. Therefore, the net charge of the NCR molecule is believed to be a key factor in the antimicrobial activity. The broad antimicrobial activity of NCR peptides also affects *S. meliloti*, which is one of the symbiotic partner rhizobia of *M. truncatula*. Indeed, some NCR peptides, including NCR035, NCR055, NCR211, and NCR247, display some inhibitory effect on the proliferation of *S. meliloti*^2,9,15^. However, NCR peptides do not actually kill rhizobia during the symbiotic process; therefore, their real concentrations *in planta* must be lower than those in in vitro experimental conditions^16,17^. To date, it remains unclear how the antimicrobial activity of NCR peptides contributes to bacteroid differentiation^18^.

NCR peptides have four or six cysteine residues at conserved positions, which are thought to form intramolecular disulfide linkages *in planta*^19^. To date, only two NCR peptides with four cysteine residues have been subjected to structural studies. One of them, NCR044, is the only NCR peptide whose three-dimensional structure has been solved^20^. The solution NMR structure of NCR044 has a short α-helix and an antiparallel β-sheet at the C-terminal region, although the N-terminal half is disordered. The disulfide linkages in NCR044 expressed in *Pichia pastoris* were C1–C4 and C2–C3, and they contributed to conformational stability. The other one, NCR247, has been proposed to have disulfide bonds at C1–C2 and C3–C4^21^, unlike NCR044. Moreover, the disordered conformation of NCR247 suggested that the disulfide linkages do not contribute to peptide folding, although they contribute to protection from proteases^22^. In addition, the disulfide linkages of NCR247 are not essential for translational inhibition and antimicrobial activity^22^. Based on these results, the structural features and the roles of disulfide bonds in NCR044 and NCR247 seem different. Limited information about the structure–function relationship of NCR peptides prevents elucidation of the biological significance of the conserved cysteine residues.

NCR169, an essential factor for bacteroid differentiation, is a cationic peptide (pI = 8.45) composed of 38 amino acid residues^8^. NCR169 is known to localize in the peribacteroid space between the bacteroid membrane and symbiosome membrane^8^. Thus, NCR169 in the peribacteroid space is thought to play essential roles in bacteroid differentiation. However, NCR169 has also been detected in isolated bacteroid extracts^2,23^. In addition, the *M. truncatula Δncr169* mutant induces early senescence of the nodule^8^. Therefore, NCR169 may have other roles aside from bacteroid differentiation. Since all four cysteine residues of NCR169 are necessary for its function *in planta*, the disulfide bonds of NCR169 are believed to be involved in its function^8^. However, the structure of NCR169 is currently unknown.

In this study, we characterized NCR169 to clarify the structure–function relationship for further understanding of the NCR family peptides. We prepared two species of NCR169 with different disulfide linkage patterns and solved their solution NMR structures. The C-terminal regions of both structures have a short antiparallel β-sheet that is similar to the folded region of NCR044. Moreover, we demonstrated that the solvent-exposed positively charged Lys-cluster of NCR169 has membrane binding ability and antimicrobial activity. Finally, we observed morphological changes in *E. coli* and *S. meliloti* caused by an NCR169-derived peptide. Our results provide new insights into the structure and function of NCR peptides.

## Results

### Preparation of NCR169

**Figure 1:**
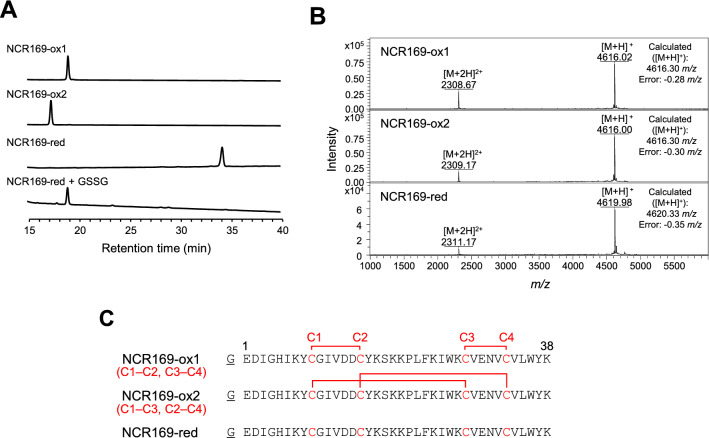
Sample preparation of NCR169 peptides. (**A**) High-performance liquid chromatography (HPLC) analyses of NCR169 peptides. Purified NCR169-ox1, NCR-ox2, NCR169-red, and reoxidized NCR169-red (NCR-red + GSSG) were analyzed by HPLC with a linear gradient of 30 to 45% acetonitrile and monitored at 220 nm. (**B**) MALDI-TOF–MS spectra of HPLC-purified NCR169-ox1 (upper), NCR169-ox2 (middle), and NCR169-red (lower). (**C**) Amino acid sequences of NCR169 peptides used in this study. The cysteine numbers (C1 to C4) are indicated in red on the sequence. The experimentally determined disulfide linkage patterns are indicated by connecting lines on the sequences. The first N-terminal residue, G (underlined), is the extra residue remaining after tag cleavage.

It has been reported that physiologically active plant defensin with disulfide bonds can be expressed in *E. coli* as a fusion protein with thioredoxin^24^. Thus, we prepared recombinant *M. truncatula* NCR169 according to the protocol because NCR peptides have been classified as a “defensin-like” family^16^. After successful expression of the fusion protein and cleavage of the thioredoxin-tag, high-performance liquid chromatography (HPLC) purification unexpectedly resulted in two peaks (major and minor), both of which showed the mass of the NCR169 oxidized form (NCR169-ox, Fig. 1A,B). To determine the disulfide linkage patterns of the two NCR169-ox peptides, protease digestion under non-reducing conditions was performed, followed by matrix-assisted laser desorption-ionization mass spectrometry (MALDI-TOF–MS) analyses (Supplementary Figs. S1 and S2). The disulfide linkage patterns were confirmed as C1–C2 and C3–C4 for NCR169-ox1 (major), and C1–C3 and C2–C4 for NCR169-ox2 (minor) (Fig. 1C). NCR169 with a C1–C4 and C2–C3 linkage pattern was not detected. HPLC analysis revealed a peak with a mass corresponding to the reduced form (NCR169-red) that showed a different retention time from the two NCR169-ox peaks (Fig. 1A,B). As an additional experiment, NCR169-red was reoxidized with oxidized glutathione (GSSG). HPLC analysis showed only one peak corresponding to NCR169-ox1 (Fig. 1A). Our results suggest that NCR169-ox1 has the most stable folding among all possible oxidized forms.

### Salt and pH stability of NCR169

Because the HPLC retention time of NCR169-red was longer than that of the NCR169 oxidized forms (Fig. 1A), the molecular surface of NCR169-red was more hydrophobic than that of the NCR169 oxidized forms. This suggested that NCR169-red is relatively salt-sensitive. To confirm it, we examined the solubility of NCR169 in various solvents, HEPES (salt-free), HEPES with salt (50 to 200 mM NaCl or KCl), and PBS. NCR169-ox1 and -ox2 were soluble in all conditions, while NCR169-red was insoluble in all conditions except the salt-free buffer (Supplementary Fig. S3). The results clearly indicated that NCR169-red is hardly a solute under physiological conditions. In other words, the disulfide bonds of NCR169 contribute to its solubility.

We further measured the circular dichroism (CD) spectra of NCR169-ox1 and -ox2 under various pH conditions to confirm their structural stability against pH (Supplementary Fig. S4). In the pH range of 2.2 to 7.0, both oxidized forms showed very small differences in the CD data. The results suggested that both peptides retained their conformations under acidic and neutral conditions. Because acidic pH is preferable for amide proton detection in NMR experiments, we performed the following solution NMR study at lower pH.

### The solution NMR structure of NCR169

**Figure 2:**
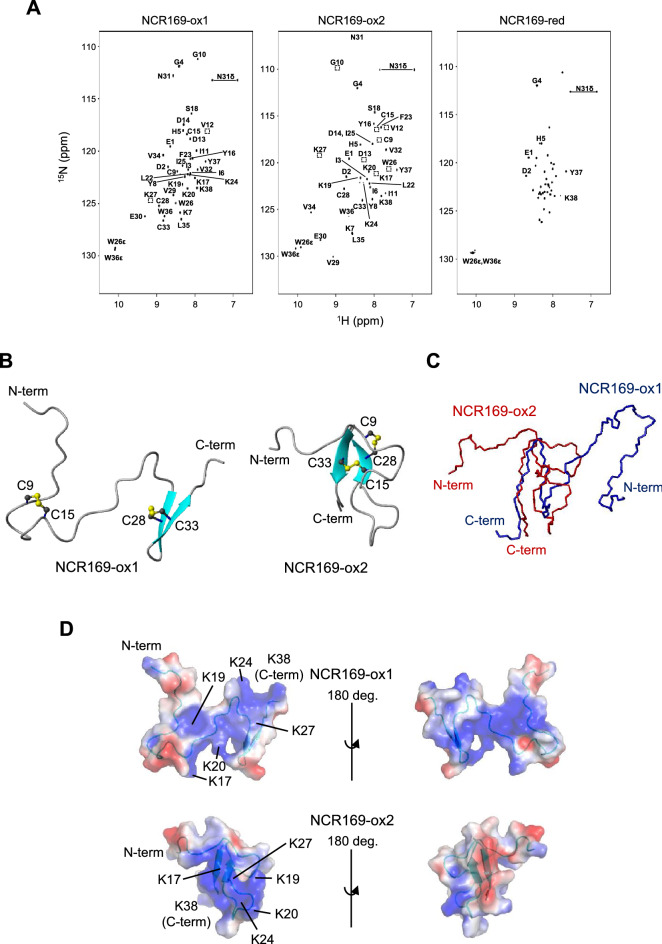
Solution NMR structures of NCR169 oxidized forms. (**A**) ^1^H–^15^N heteronuclear single quantum coherence (HSQC) spectra of NCR169-ox1 (left), NCR169-ox2 (middle), and NCR169-red (right). In the NCR169-ox1 and NCR169-ox2 spectra, signals are labeled with amino acid type and residue number. In the NCR169-red spectrum, only the signals predicted from other spectra are labeled. (**B**) Ribbon models of NCR169-ox1 (left) and NCR169-ox2 (right). Two pairs of disulfide linkages are depicted in the ball-and-stick model. The secondary structural regions were estimated using the MOLMOL program. (**C**) Structural overlay of NCR169-ox1 (blue) and -ox2 (red). Only their backbones are depicted. (**D**) The electrostatic distribution was plotted on the molecular surface of NCR169-ox1 (upper) and NCR169-ox2 (lower). The negative, positive, and hydrophobic surfaces are shown in red, blue, and white, respectively. The lysine residues forming a positively charged cluster are labeled.

To obtain structural insights into NCR169, we performed NMR analyses of the two oxidized forms (NCR169-ox1 and -ox2) and the reduced form (NCR169-red) in the absence of salt. For the NMR samples, stable isotope (^15^N)-labeled peptides were prepared for the three NCR169 species. In the ^1^H–^15^N HSQC spectra of NCR169-ox1 and -ox2, well-dispersed peaks were observed, suggesting that the peptides have a secondary structure (Fig. 2A). Moreover, the two spectra were different, indicating that they formed different conformations. In contrast, the NMR spectrum of NCR169-red showed poorly dispersed peaks, suggesting a disordered structure (Fig. 2A). The NMR data indicated that the disulfide linkages of NCR169 are necessary for folding. Further NMR analysis successfully revealed the three-dimensional structures of NCR169-ox1 and -ox2 (Fig. 2B and Supplementary Fig. S5). The structural characteristics are summarized in Supplementary Table S1. NCR169-ox1 only has an anti-parallel β-sheet composed of two strands at the C-terminal region (residues 27–29 and 32–34). These β-strands were in agreement with the predictions from the secondary chemical shifts (Supplementary Fig. S6A). Intriguingly, the secondary structure of NCR169-ox1 was identical to that of NCR169-ox2, although their disulfide patterns were completely different (Fig. 2B). The chemical shift differences between NCR169-ox1 and -ox2 revealed structural differences derived from different disulfide linkage patterns (Supplementary Fig. S6B). As shown in Fig. 2B,C a major difference between the two structures was found in the position of the unstructured N-terminal region. In NCR169-ox1, the position of the extended N-terminal region is undefined, while that of NCR169-ox2 is relatively fixed by the disulfide bonds and covers one side of the β-sheet. Structural comparison searches using the Dali server revealed that NCR169-ox1 and -ox2 structures did not match any structure in the PDB, indicating that NCR169-ox1 and -ox2 have novel structures. The C-terminal anti-parallel β-sheet in the NCR169 oxidized forms is similar to that of NCR044 (PDB ID: 6U6G)^20^. However, unlike NCR044, the NCR169 oxidized forms have no α-helix. The dynamics of NCR169-ox1 and -ox2 were assessed using *T*_1_, *T*_2_, and {^1^H}-^15^N heteronuclear Overhauser effect (hNOE) (Supplementary Fig. S7). The N-termini (residues 1 to 5) of both NCR169-ox1 and -ox2 showed relatively small values for 1/*T*_1_ (= *R*_1_), 1/*T*_2_ (= *R*_2_), and hNOE, indicating high flexibility. Overall, the flexibility of NCR169-ox1 was relatively higher than that of NCR169-ox2. One of the reasons for the difference in dynamics may be the structural dissimilarity of the disulfide bonds connecting the loop with the β-sheet.

We further investigated the electrostatic potentials on the molecular surfaces of NCR169-ox1 and -ox2 (Fig. 2D). In NCR169-ox1, hydrophobic surfaces are partially formed around the β-sheet because they are completely exposed to the solvent. On the other hand, such a hydrophobic surface is not found in NCR169-ox2 because this side of the β-sheet is masked by the N-terminal loop. In both molecules, a positively charged area exists on the molecular surface. This region contains Lys17, Lys19, Lys20, Lys24, and Lys27, which are located in the middle of the primary structure. Since NCR169 is unstructured, we also expect the Lys-rich region of NCR169-red to be exposed to the solvent.

### NCR169 binds to liposomes

**Figure 3:**
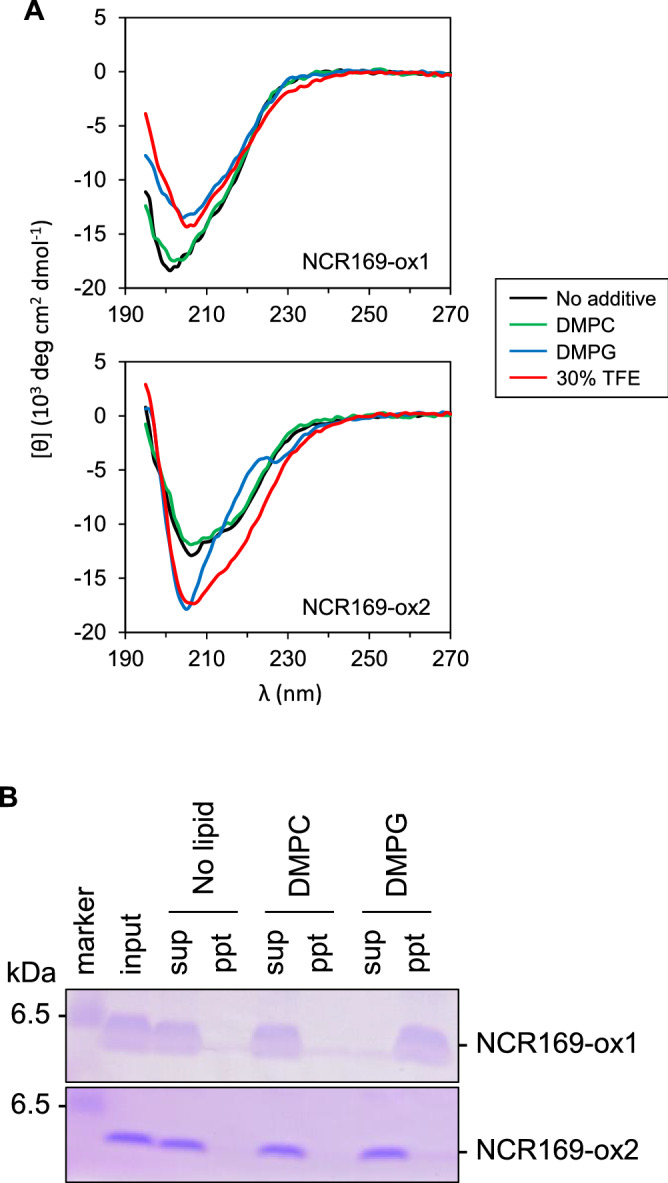
NCR169 binds to the bacterial phospholipid. (**A**) Circular dichroism (CD) spectra of NCR169 peptides (NCR169-ox1 and -ox2). NCR169 peptides (20 μM) were dissolved in the following solvents: water (No additives), 2 mM DMPC liposomes, 2 mM DMPG liposomes or 30% 2,2,2-trifluoroethanol (TFE). (**B**) The results of liposome binding assays. The NCR169 peptide (20 μM) was incubated with buffer (No lipid), 2 mM DMPC liposomes, or 2 mM DMPG liposomes. After incubation at room temperature for 30 min, the mixture was ultra-centrifuged at 200,000×*g*. The supernatant and pellet fractions were analyzed by Tricine-SDS-PAGE. The no lipid sample before ultra-centrifugation was used as the input.

Because NCR169 has a positively charged area on the molecular surface, we expected that NCR169 would interact with the negatively charged bacterial cell membrane. Thus, we performed experiments using natural dimyristoylphosphatidylcholine (DMPC) and anionic dimyristoylphosphatidylglycerol (DMPG), which are components of the cell membrane. First, the CD spectra were measured to monitor the conformational changes of the NCR169 oxidized forms under various conditions (Fig. 3A). The spectrum of NCR169-ox1 remained almost unchanged with the addition of DMPC liposomes, whereas the addition of DMPG liposomes resulted in a slightly different spectrum, which was very similar to that in 30% 2,2,2-trifluoroethanol (TFE). Similar to NCR169-ox1, although the CD spectrum of NCR169-ox2 was unaffected by DMPC, it was affected by DMPG. The CD spectrum of NCR169-ox2 with DMPG was slightly different from that in 30% TFE. In both NCR169-ox1 and -ox2, the effect of DMPG addition was greater than that of pH change (Supplementary Fig. S4). However, for both peptides, the CD signals at 208 and 222 nm as the α-helix indicator and at 218 nm as the β-sheet indicator were hardly affected by the addition of DMPG. The results suggested that the secondary structures of NCR169-ox1 and -ox2 are retained even when interacting with DMPG, meaning that additional secondary structures are not induced by the interaction.

Next, we examined whether NCR169 binds to phospholipid liposomes. Neither NCR169-ox1 nor -ox2 bound to the DMPC liposomes (Fig. 3B), and only NCR169-ox1 bound to the DMPG liposomes. Because phosphatidylglycerol (PG), an anionic lipid, is known to be the main component of the cell membrane of gram-negative bacteria^25^, the results indicated that NCR169-ox1 may bind to the cell membrane of gram-negative bacteria, including rhizobia. This result implies that NCR169-ox1 has feasible disulfide linkage pairs *in planta*.

### NCR169 shows antimicrobial activity

**Figure 4:**
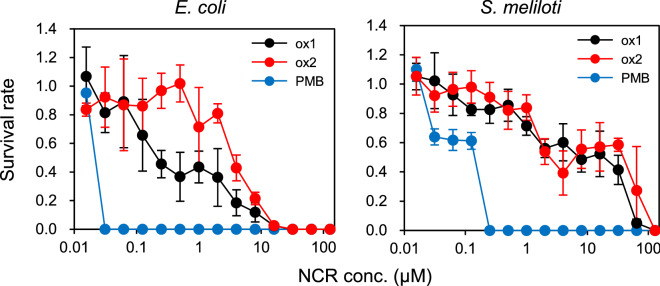
*M. truncatula* NCR169 has antimicrobial activity against *Escherichia coli* K-12 and its symbiotic partner *Sinorhizobium meliloti*. Bacteria (*S. meliloti* and *E. coli* K-12) were treated with various NCR169 peptide (NCR169-ox1 and -ox2) and PMB concentrations, and survival rates were calculated based on untreated bacteria. All experiments were repeated at least four times. Error bars represent standard deviation (SD).

In the above section, we suggested that NCR169 is likely to interact with the cell membrane of gram-negative bacteria. This means that, although NCR169 is believed to lack antimicrobial activity, NCR169 may have potential antimicrobial activity against these bacteria^14^. Thus, we experimentally examined the effects of NCR169-ox1 and -ox2 on the proliferation of *E. coli* and *S. meliloti*, a symbiotic partner of *M. truncatula*. Polymyxin B (PMB) was used as the positive control. As shown in Fig. S8, the two NCR169 oxidized forms displayed antimicrobial activity against both *E. coli* and *S. meliloti*. Furthermore, the survival rates of both bacteria decreased with increasing concentrations of the two NCR169 peptides (Fig. 4). However, *E. coli* was more sensitive to the peptides than *S. meliloti*. Based on the IC_50_, the antimicrobial activity of NCR169-ox1 was higher than that of NCR169-ox2 (Supplementary Table S2). In the above section, we already found that NCR169-ox1 binds to phospholipids, whereas NCR169-ox2 hardly binds. Taken together, the results suggested that binding of NCR169 to the bacterial cell membrane is likely related to its antimicrobial activity.

### The Lys-rich region is responsible for the antimicrobial activity

**Figure 5:**
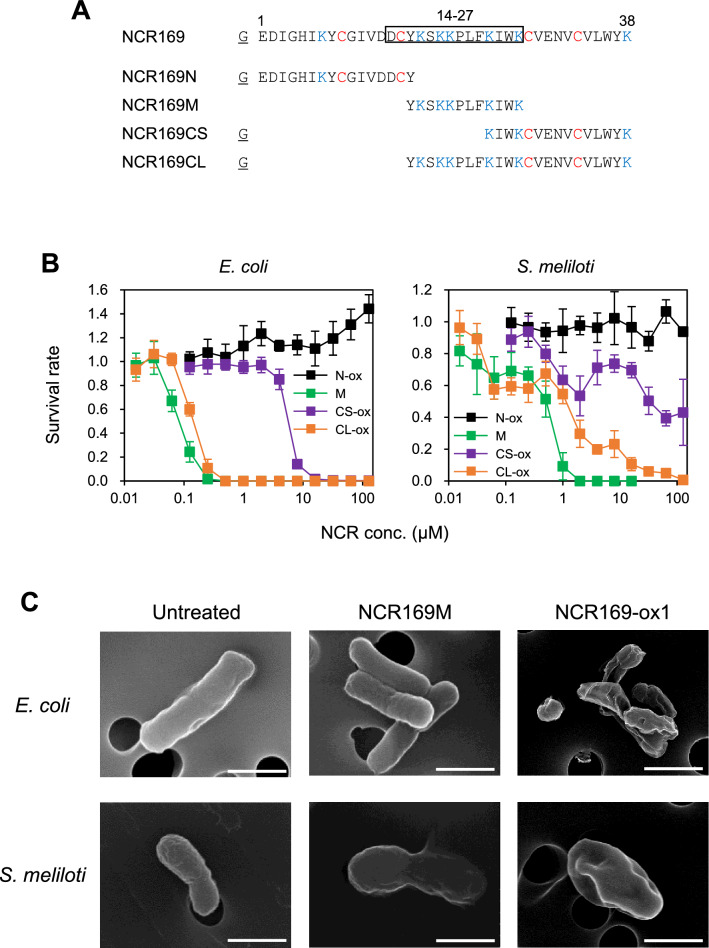
The central Lys-rich region of NCR169 is the determinant of antimicrobial activity. (**A**) Amino acid sequences of NCR169 and NCR169-derived peptides (NCR169N, NCR169M, NCR169CS, and NCR169CL) in this study. The boxed range (positions 14 to 27) indicates the predicted antimicrobial region. Lysine and cysteine residues are shown in blue and red, respectively. The first N-terminal residue, G (underlined), is the extra residue remaining after tag cleavage. (**B**) Antimicrobial activities of NCR169-derived peptides (NCR169N-ox, NCR169M, NCR169CS-ox, and NCR169CL-ox). Bacteria (*E. coli* K-12 and *S. meliloti*) were treated with various peptide concentrations, and survival rates were calculated based on untreated bacteria. All experiments were repeated at least four times. Error bars represent standard deviation (SD). (**C**) Scanning electron microscopy (SEM) images of *E. coli* and *S. meliloti* cells untreated or treated with NCR169. All treatments were performed at IC_100_ for 24 h. (Scale bars: 1 μm).

We attempted to identify the region responsible for the antimicrobial activity in the amino acid sequence of NCR169. The prediction tool named collection of anti-microbial peptides (CAMP) suggested that the region from residues 14 to 27 containing several lysine residues could have antimicrobial activities (Supplementary Fig. S9). Therefore, based on this prediction, we prepared four kinds of NCR169-derived peptides for antimicrobial activity measurements (Fig. 5A). MALDI-TOF–MS results indicated that peptides containing two cysteine residues were obtained in oxidized forms (NCR169N-ox, NCR169CS-ox, NCR169CL-ox) (Supplementary Fig. S10A). Furthermore, CD spectra suggested that NCR169N-ox and NCR169M have random structures, whereas NCR169CS-ox and NCR169CL-ox contain β-sheets (Supplementary Fig. S10B and S11A). The structural features of these peptides were identical to those of the corresponding regions in the intact peptide.

Antimicrobial tests against *E. coli* and *S. meliloti* were performed using various concentrations of the NCR169-derived peptides. The antimicrobial activity profile of each NCR169-derived peptide against *E. coli* was similar to that against *S. meliloti*, but *E. coli* was generally more sensitive to the peptides than *S. meliloti* (Fig. 5B and Supplementary Table S2). NCR169M and NCR169CL-ox had stronger activity than NCR169CS-ox. This result suggested that the region corresponding to NCR169M containing lysine residues contributes the most to the antimicrobial activity, as predicted. This finding was confirmed by the observation that NCR169N-ox, which does not contain the region corresponding to NCR169M, had no antimicrobial activity.

NCR169M showed the highest antimicrobial activity against the symbiotic partner, *S. meliloti*. The order of antimicrobial potency was estimated as follows: NCR169M > NCR169CL (NCR169M + C-terminal region) > NCR169-ox1 (N-terminal region + NCR169M + C-terminal region). Thus, both the N-terminal flexible loop and C-terminal β-sheet appear to have some impairing effect on antimicrobial activity.

We further confirmed whether NCR169-derived peptides bind to phospholipid liposomes. As expected, NCR169M, NCR169CS-ox, and NCR169CL-ox bound to DMPG liposomes, but NCR169N-ox did not (Supplementary Figs. S11B and S12). This result suggested that the region corresponding to NCR169M, at least residues 24 to 27, is involved in binding to DMPG. As described in the previous section, the secondary structure of NCR169-ox1 was unchanged by DMPG binding (Fig. 3A). Thus, we concluded that the NCR169M corresponding part of NCR169-ox1 binds to the cell membrane of gram-negative bacteria in an identical manner, without secondary structure formation. In general, antimicrobial peptides are known to act on bacterial membranes by forming the amphipathic α-helix^26^. Thus, the disordered conformation of the central Lys-rich region of NCR169 is a unique property that is key to membrane binding and antimicrobial activity.

### Morphology of bacteria treated with the NCR169 Lys-rich region

We further observed bacterial cells using scanning electron microscopy (SEM) to investigate the morphological effects of the NCR169 Lys-rich region (NCR169M) and NCR169-ox1. *E. coli* cells treated with either NCR169M or NCR169-ox1 for 5 min and 2 h were similar to those of untreated cells, but cell aggregation was observed after 24 h (Fig. 5C and Supplementary Fig. S13). Compared to untreated *S. meliloti* cells, cell swelling was observed in PMB-treated cells (Supplementary Fig. S13). This morphological change is consistent with a report by Mikulass et al.^15^. In contrast, NCR169M- and NCR169-ox1-treated *S. meliloti* cells did not show such a change. *S. meliloti* cells treated with NCR169M for 5 min and 2 h were indistinguishable from untreated cells (Supplementary Fig. S13). After 24 h, the cells were enlarged, and the cell surface became smooth (Fig. 5C and Supplementary Fig. S13). However, unlike *E. coli* treated with NCR169M and NCR169-ox1, no cell aggregation was observed in *S. meliloti* treated with NCR169M or NCR169-ox1. These results suggest that the effects of NCR169 differ between *E. coli* and *S. meliloti*, a symbiotic partner of *M. truncatula*. No protrusions were observed on the cell surfaces of either *E. coli* or *S. meliloti* after NCR169M and NCR169-ox1 treatments. Our SEM results showed that the morphological effects of NCR169M on *E. coli* and *S. meliloti* were identical to those of NCR169-ox1. These findings are consistent with previous observations of *S. meliloti* treated with NCR169^4^. It has been reported that high cationic NCR peptides (pI > 9) cause protrusions on the cell surface of bacteria such as *E. coli* and *S. meliloti*^4,12,13^. Therefore, we suggest that the effect of NCR169 on *E. coli* and *S. meliloti* is different from that of NCR247 and NCR335.

## Discussion

### Native conformation of NCR169 in planta

The native conformation of NCR169 *in planta* is still unknown because the amount of NCR169 in roots is too small to be purified for analysis. The finding that NCR169 in the reduced state is unstable under physiological salt conditions supports the idea that native NCR169 presumably forms intramolecular disulfide bonds, just as other defensin family members. This idea is consistent with the known fact that all four cysteine residues of NCR169 are necessary for the maintenance of its function *in planta*^8^. In this work, we successfully present three pieces of experimental results that suggest the native conformation of NCR169 *in planta,* although three disulfide linkage patterns are possible. First, NCR169-ox1 was identified as the major component when NCR169 was expressed in *E. coli*. Second, reoxidation of reduced NCR169 yielded only NCR169-ox1. This indicated that NCR-ox1 was the most stable oxidized form. Third, only NCR169-ox1 bound to negatively charged DMPG liposomes. Thus, we propose that NCR169 with C1–C2 and C3–C4 disulfide linkage patterns is the most likely conformation *in planta.*

Before the present study, there were only proposed disulfide patterns for two NCR peptides with four cysteine residues, NCR044 and NCR247, whose disulfide linkage patterns are not identical; C1–C2 and C3–C4 for NCR247, and C1–C4 and C2–C3 for NCR044^20,27^. Here, we reveal that NCR169 has a C1–C2 and C3–C4 disulfide linkage pattern, which is identical to that of NCR247, but different from that of NCR044. Thus, NCR peptides with four cysteine residues could be categorized into subgroups based on their disulfide linkage patterns.

### Structure–function relationship of NCR169

The antimicrobial activity and phospholipid binding of NCR169-ox1 were superior to those of NCR169-ox2. Compared to NCR169-ox2, NCR169-ox1 has a functional Lys-rich region with higher solvent accessibility and flexibility (Supplementary Fig. S7). Therefore, these properties are conceivable key factors in the action of the Lys-rich region.

The structural features of NCR169-ox1 are close to those of NCR044, except that NCR169-ox1 has no α-helix^20^. Both structures have largely disordered N-terminal regions and an anti-parallel β-sheet in the C-terminal regions. Such disordered features are also found in all oxidized forms of NCR247^22^. Therefore, based on the available structural information, it seems that the disordered structure is a common feature of the three NCR peptides. Intrinsically disordered proteins (IDPs) and IDP regions are known to be able to interact with many biomolecules due to their flexibility^28,29^. Therefore, the disordered regions of NCR peptides may recruit their specific targets. It is worth noting that the NCR169 homolog peptides found in *Medicago sativa* and *Melilotus albus* also have a Lys-rich region in the middle part of their sequences^8^. Therefore, the flexible Lys-rich region may potentially exert NCR169-specific functions.

NCR peptides are classified as defensin-like proteins. A representative defensin structure is composed of a double- or triple-stranded β-sheet and typically, an α-helix^30,31^. Normally, the loop between β-strands is responsible for the function of the defensin^30^. Although the characteristic β-sheet structure of NCR169-ox is somewhat similar to that of common defensins, the loop between the two strands seems to be too short to show defensin activity.

### Antimicrobial activity of NCR169

A simple model peptide epsilon-poly-lysine (ε-PL), which is positively charged, is known to have antimicrobial activity^32^. The activity depends on the number of lysine residues (n), and the activity is shown when n > 10^33^. It is also known that the positive net charge (+ 1 to + 5) of antimicrobial peptides is related to the activity, such as the ability to induce membrane depolarization and disruption^30^. The net charge of NCR169 is calculated as + 2, which is mainly due to the central Lys-rich region responsible for the antimicrobial activity against *E. coli* and *S. meliloti*. Thus, the antimicrobial activity of NCR169 could be derived from direct charge interaction between the cationic Lys-rich region and the anionic bacterial cell membrane of bacteria^26^. The antimicrobial activity (IC_50_) of the central Lys-rich region in NCR169 was of the same order as that of PMB. Therefore, NCR169 may be a useful candidate as an antimicrobial agent.

In this study, *E. coli* cell aggregation was observed upon NCR169 treatment. These changes are similar to those caused in *E. coli* by human α-defensin 5 (HD5)^34^. HD5 is known to bind to lipopolysaccharide (LPS), the outer membrane component of gram-negative bacteria^35^. Therefore, NCR169 may have some effect on LPS.

At present, the significance of the weak antimicrobial activity of NCR169 against *S. meliloti in planta* remains unclear. Since the concentration of NCR peptides *in planta* is believed to be very low compared to that in in vitro assays^16,17^, it is likely that NCR169 has a bacteriostatic action, rather than bactericidal, against rhizobia. The weak antimicrobial activity of NCR169 might be a factor that regulates the differentiation time of rhizobia. Further research on NCR169 and other NCR peptides is required to decipher the structure–function relationship of the NCR family.

## Materials and methods

### NCR169 peptides

The custom-made plasmid pET32b-NCR169, which contains the DNA encoding NCR169 in the pET32b(+) vector, was purchased from GenScript (Tokyo, Japan). The plasmid was introduced into Rosetta-gami B (DE3) pLysS *E*. *coli* cells (MilliporeSigma, Burlington, MA, USA). *E*. *coli* cells with the plasmid were cultured in Luria broth (LB)/Ampicillin (Amp) medium at 37 °C, and 0.1 mM isopropyl β-d-1-thiogalactopyranoside (IPTG) was added when the optical density at 600 nm (OD_600_) reached 0.6. After overnight (12 to 16 h) incubation at 37 °C, the cells were collected and stored at − 30 °C until use. The NCR169 peptide was expressed as a fusion protein with thioredoxin (Trx) and a His_6_-tag, which can be removed using the tobacco etch virus (TEV) protease. For ^15^N isotopic labeling, *E*. *coli* cells were cultured in M9/Amp medium at 37 °C, and 0.1 mM IPTG was added when the OD_600_ reached 0.6. After 22 h of incubation at 37 °C, the cells were collected and stored at − 30 °C until use. *E*. *coli* cells were suspended in phosphate-buffered saline (PBS) containing 1 mM phenylmethylsulfonyl fluoride (PMSF), sonicated, and centrifuged at 10,000×*g* for 15 min. After centrifugation, the supernatant was collected, and the fusion protein was purified using a TALON column (Takara Bio, Kusatsu, Japan). Protein concentration was estimated using a bicinchoninic acid (BCA) assay kit (Thermo Fisher Scientific, Waltham, MA, USA). TEV protease (Sigma-Aldrich, St. Louis, MO) was added to the purified fusion protein (50 units TEV protease/1 mg protein), and the mixture was incubated at 30 °C for 20–24 h. The resulting peptides were purified using a high-performance liquid chromatography (HPLC) system (Shimadzu 10A VP, Kyoto, Japan or Thermo Fisher Scientific UltiMate 3000, Waltham, MA, USA) with a C18 analytical column (Protein-R, 4.6 ID × 250 mm; Nacalai Tesque, Inc., Kyoto, Japan) at a flow rate of 0.5 mL/min. A gradient condition was configured at 30–32.5% acetonitrile (ACN) for 30 min, followed by a washing step for 15 min. The eluate was monitored at a wavelength of 220 nm. Fractions containing NCR169 peptides were collected and dried under vacuum conditions. The purified peptides were assessed using MALDI-TOF–MS (ultrafleXtreme; Bruker, Billerica, MA, USA).

To prepare the reduced peptide (NCR169-red), NCR169-ox was reduced with 10 mM tris(2-carboxyethyl)phosphine (TCEP) at pH 10.5, followed by acidification using trifluoroacetate (TFA) and HPLC purification with a C18 column and 30–45% ACN gradient. The fractions were collected and dried under vacuum conditions. The purified peptides were confirmed using MALDI-TOF–MS.

### Disulfide linkage determination

NCR169-ox1 was digested with Lys-C under non-reducing conditions. The digests were subjected to MALDI-TOF–MS, and the resulting spectra were analyzed using BioTools (Bruker, Billerica, MA, USA) and PeptideMass (https://web.expasy.org/peptide_mass/). To determine the disulfide linkage of NCR169-ox2, a three-step digestion using Lys-C, Glu-C, and Asp-N was performed.

### NCR169-derived peptides

Each custom-made plasmid, pET32b-NCR169N, pET32b-NCR169CS, and pET32b-NCR169CL, was transformed into Rosetta-gami B (DE3) pLysS *E*. *coli* cells. The tag-fused proteins were expressed by the addition of 0.1 mM IPTG and purified using a TALON column. After tag cleavage using TEV protease, the resulting peptides were purified using an HPLC system with a C18 column and 27.5–32.5% ACN gradient. The purified peptides were confirmed using MALDI-TOF–MS. The chemically synthesized NCR169M peptide was purchased from Scrum Inc. (Tokyo, Japan).

### NMR measurements and structural calculations

The NMR samples were dissolved in 10% or 100% D_2_O (pH 2.2). NMR experiments were performed on a Bruker AVANCE III 800 spectrometer at a ^1^H resonance frequency of 800.01 MHz. All data were recorded with a TCI cryogenic probe at 25.0 °C. The FID data were processed with NMRPipe^36^ and analyzed using Sparky^37^. ^1^H and ^15^N chemical shifts were assigned using ^1^H–^1^H TOCSY, ^1^H–^1^H NOESY, ^1^H–^15^N-HSQC, ^15^N-edited TOCSY, and ^15^N-edited NOESY spectra. All NOESY experiments were performed with a mixing time of 300 ms. The secondary chemical shifts (Δδ) were calculated as follows: Δδ = δ_observed_ − δ_random coil_. The random coil value (δ_random coil_) reported by Wishart et al. was used for the calculation^38^. The following formula was used to calculate the chemical shift difference.

$$ \Delta \delta_{{{}^{1}H,{}^{15}N}} = \sqrt {\left( {\Delta \delta_{{{}^{1}H}} } \right)^{2} + \frac{1}{15}\left( {\Delta \delta_{{{}^{15}N}} } \right)^{2} } $$

The three-dimensional structures of the NCR169 oxidized forms were calculated using CYANA^39^. Nuclear Overhauser effects (NOEs) were used as constraints for the structure calculation. The hydrogen bond constraints were introduced based on the proximity in early structure calculations and the secondary structure predicted by secondary chemical shifts. The dihedral angles predicted by the TALOS + server (https://spin.niddk.nih.gov/bax/nmrserver/talos/) were used as constraints for the structure calculation^40^. The structural figures and electrostatic potentials were generated using the MOLMOL and PyMOL programs, respectively^41,42^. A three-dimensional structure comparison search was performed using the Dali server^43^. To characterize the backbone dynamics, *T*_1_, *T*_2_, and {^1^H}-^15^N NOE experiments were performed^44^. For *T*_1_ measurements, the relaxation delays were 10, 26, 50, 82, 138, 306, 410, 502, 700, 853, 1100, and 1300 ms. For *T*_2_ measurements, the relaxation delays were 8.7, 15, 22, 33, 43, 70, 88, 104, 130, 156, 170, 200, 255, and 400 ms. *T*_*1*_ and *T*_*2*_ values were determined by fitting the measured peak intensities to a two-parameter function as follows:

$$ I\left( t \right) = I_{0} \exp \left( { - \frac{t}{{T_{1,2} }}} \right) $$

where *I*(*t*) and *I*_0_ are the intensities after a delay time *t* and at time *t* = 0, respectively^44^. The recycling delay in the {^1^H}-^15^N NOE experiments was set to 5 s. The {^1^H}-^15^N NOE experiments were repeated twice. The chemical shifts of NCR169-ox1 and -ox2 were deposited in BMRB with IDs 36,362 and 36,363, respectively. The structures of NCR169-ox1 and -ox2 were deposited in PDB with IDs 7CKD and 7CKE, respectively.

### Liposome preparation

The phospholipids DMPC and DMPG were purchased from Avanti Polar Lipids (Alabaster, AL, USA). An appropriate amount of phospholipid was dissolved in chloroform and dried under vacuum for 1 h. The lipid film was suspended in water, vortexed, and sonicated for 30 min to form the liposomes.

### Circular dichroism

CD spectra were recorded within a wavelength range of 270–195 nm using a JASCO J-820 spectropolarimeter (JASCO International Co., Ltd., Tokyo, Japan). A quartz cell with a light path length of 1 mm was used for the measurements. The sample temperature was maintained at 25 °C during the measurements.

### Liposome sedimentation assays

The assay was carried out as described previously^45^. Briefly, equal volumes of NCR169 peptide (20 μM) and liposomes (2 mM) were mixed in assay buffer (20 mM HEPES, 150 mM NaCl, pH 7.0) and incubated for 30 min at room temperature, followed by ultracentrifugation at 200,000×*g* for 15 min at 20 °C. After centrifugation, the supernatant was collected, and the pellet was dissolved in the same volume of assay buffer containing 0.2% Triton X-100. The supernatant and pellet fractions were subjected to Tricine-SDS-PAGE, and peptides were detected by Coomassie Brilliant Blue staining.

### Antimicrobial peptide prediction

To predict the antimicrobial region of NCR169, we used the Predict Antimicrobial Region within Peptides tool from CAMP^46^. The peptide length was set to 12, and all four algorithms were selected for the prediction. For the prediction with CAMP, four algorithms, support vector machine (SVM), random forest (RF), artificial neural network (ANN), and discriminant analysis (DA) were selected. Three algorithms (SVM, RF, and DA) provided the antimicrobial peptide (AMP) or non-antimicrobial peptide (NAMP) decision and AMP probability. The ANN algorithm provided only the AMP or NAMP decision. For the ANN algorithm that provided no AMP probability, AMP and NAMP were set to 1 and 0, respectively.

### Antimicrobial tests

Antimicrobial tests were performed using the drop plate method^12,47^. Bacterial strains in the logarithmic phase (OD_600_ = 0.5–0.8) were diluted with 5 mM HEPES–KOH (pH 7.0) to a bacterial culture value of 1 × 10^6^ CFU/mL. At OD_600_ = 0.1, the concentrations of *S. meliloti* and *E. coli* K-12 cells were 6.4 × 10^6^ CFU/mL and 5.2 × 10^7^ CFU/mL, respectively. Equal volumes of bacteria (*S. meliloti* or *E. coli* K-12) and NCR169 peptide or PMB were mixed and incubated at room temperature for 2 h. For antimicrobial tests against *S. meliloti*, 10 μL of each serially diluted mixture was spotted on MHB plates with 2.5 mM MgCl_2_, and 2.5 mM CaCl_2_, followed by incubation at 30 °C for 40 h. For antimicrobial tests against *E. coli* K-12, 10 μL of each mixture was spotted on Mueller Hinton Broth (MHB; MilliporeSigma, Burlington, MA, USA) plates followed by incubation at 30 °C for 13 h.

To estimate survival rate, the peptide-treated bacterial mixtures were serially diluted with the buffer before spotting on the plate. Bacterial survival rates were calculated using the colony numbers of each bacterial strain with or without peptide treatment. All experiments were repeated at least four times.

### Scanning electron microscopy (SEM)

Bacterial strains in the logarithmic phase were diluted with 5 mM HEPES–KOH (pH 7.0) to a bacterial culture value of 1 × 10^6^ CFU/mL. Equal volumes of diluted bacterial culture and PMB or NCR169 peptide were mixed and incubated at room temperature for 5 min, 2 h, and 24 h. After incubation, the cells were fixed with 2.5% (v/v) glutaraldehyde in 0.05 M cacodylate buffer (pH 7.4) and spotted on a polycarbonate membrane filter (nano-percolator; JEOL, Tokyo, Japan). The filter was washed with 0.1 M cacodylate buffer (pH 7.4) and treated for 2 h with 1% OsO_4_ in 0.1 M cacodylate buffer (pH 7.4) for post-fixation. The samples were dehydrated with an ethanol gradient series (50, 70, 80, 90, and 100%) and twice with 100% ethanol for 10 min each. The sample was then incubated twice with 100% t-butanol for 15 min and freeze-dried (ES-2030; Hitachi, Ltd., Tokyo, Japan). After applying an 8 nm platinum coating, the samples were observed using a scanning electron microscope (S-4700; Hitachi).

## Supplementary Information

Supplementary Information.
