# Supplementary material for: Structure and antimicrobial activity of NCR169, a nodule-specific cysteine-rich peptide of Medicago truncatula
Source: Sci Rep. 2021 May 10;11:9923. doi: 10.1038/s41598-021-89485-w (PMC8110993; doi:10.1038/s41598-021-89485-w)
Supplement: Supplementary file 1 — Supplementary Information. [file 41598_2021_89485_MOESM1_ESM.pdf]

Supplementary information

Structure and antimicrobial activity of NCR169, a nodule-specific cysteine-rich peptide of *Medicago truncatula*

Noriyoshi Isozumi[1], Yuya Masubuchi[1], Tomohiro Imamura[2], Masashi Mori[2], Hironori Koga[2], and Shinya Ohki[1]*

[1]Center for Nano Materials and Technology (CNMT), Japan Advanced Institute of Science and Technology (JAIST), 1-1 Asahidai, Nomi, Ishikawa 923-1292, Japan
[2]Ishikawa Prefectural University, 1-308, Suematsu, Nonoichi, Ishikawa, 921-8836, Japan

*Corresponding author: Shinya Ohki
E-mail address: shinya-o@jaist.ac.jp

A

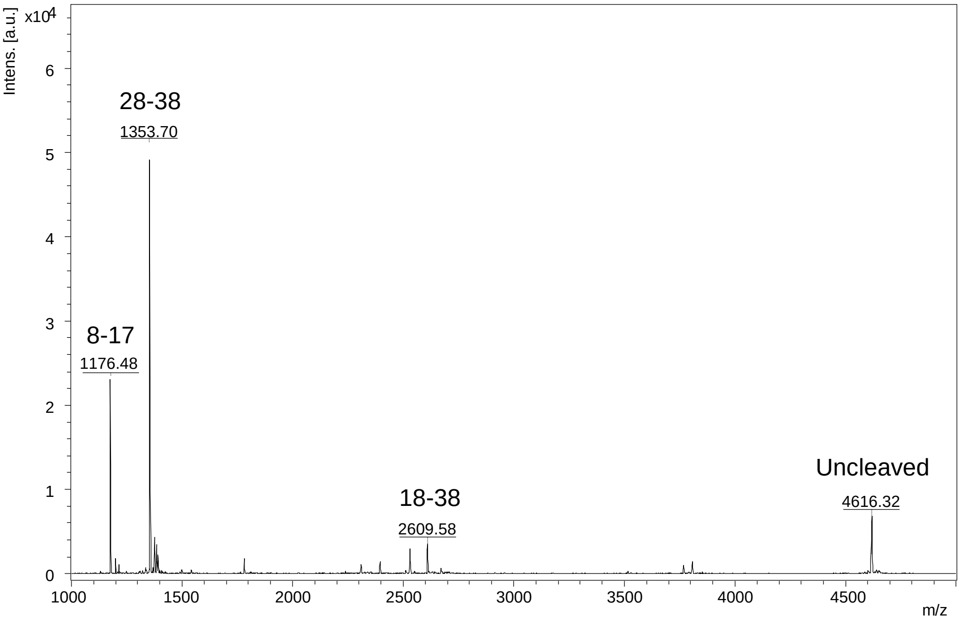

B

Position	Corresponding sequence	Calculated *m/z*	Observed *m/z*	Error (*m/z)*
8-17	YCGIVDDCYK	1176.47	1176.48	+0.01
18-38	SKKPLFKIWKCVENVCVLWYK	2609.41	2609.58	+0.17
28-38	CVENVCVLWYK	1353.63	1353.70	+0.07

Supplementary Figure S1. Disulfide linkage determination of NCR169-ox1. (A) MALDI-TOF-MS spectrum of NCR169-ox1 treated with Lys-C. (B) Assignment of NCR169-ox1 fragments detected by MALDI-TOF-MS.

A

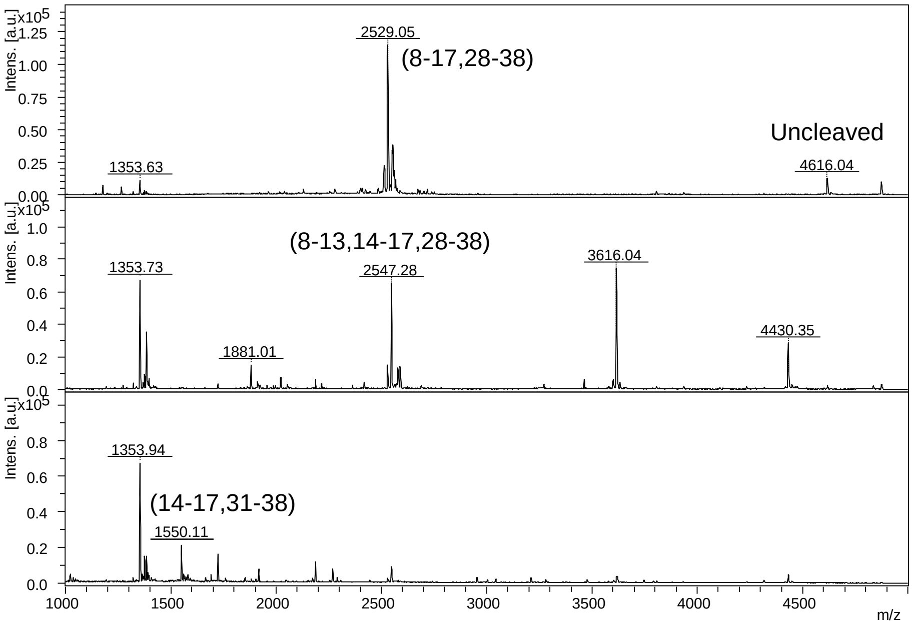

B

Position	Corresponding sequence	Calculated *m/z*	Observed *m/z*	Error (*m/z*)
(8-17,28-38)	YCGIVDDCYK CVENVCVLWYK	2529.10	2529.05	+0.05
(8-13,14-17,28-38)	YCGIVDDCYK CVENVCVLWYK	2547.11	2547.28	+0.17
(14-17,31-38)	DCYK NVCVLWYK	1549.72	1550.11	+0.39

Supplementary Figure S2. Disulfide linkage determination of NCR169-ox2. (A) MALDI-TOF-MS spectra of NCR169-ox2 after three-step digestion using Lys-C (upper), Glu-C (middle), and Asp-N (lower). (B) Assignment of NCR169-ox2 fragments detected by MALDI-TOF-MS.

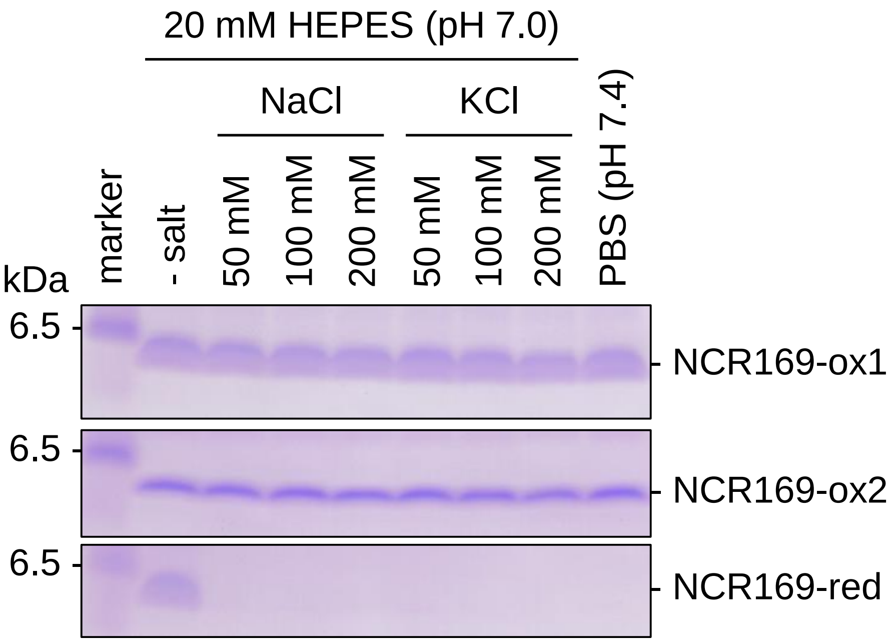

Supplementary Figure S3. Solubility assessment of NCR169. The same amount of NCR169 was dissolved in various solvents. After incubation at room temperature for 1 h, the solution was centrifuged at 20,000 × g and the supernatant was analyzed by Tricine-SDS-PAGE.

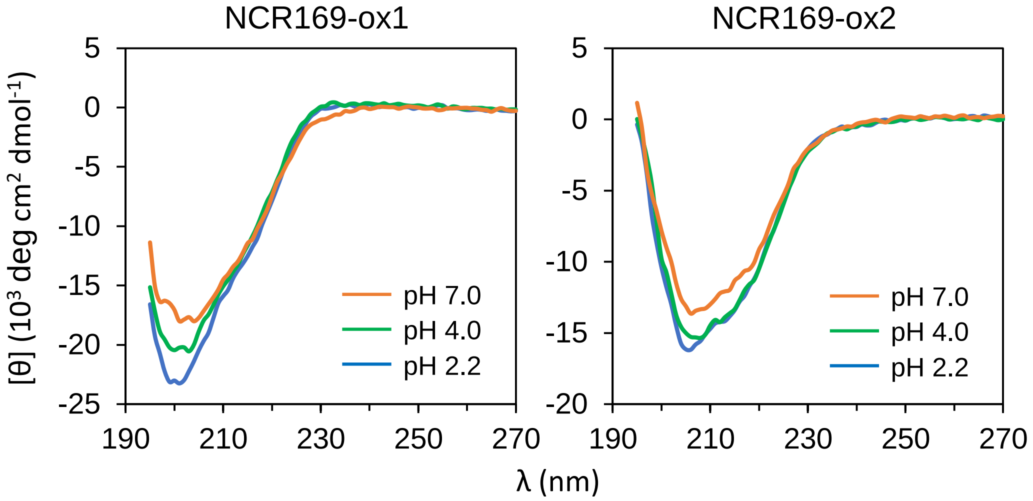

Supplementary Figure S4. Circular dichroism (CD) spectra of NCR169-ox1 and -ox2 under various pH conditions. The signal reflecting β-sheet structure (218 nm) was hardly changed.

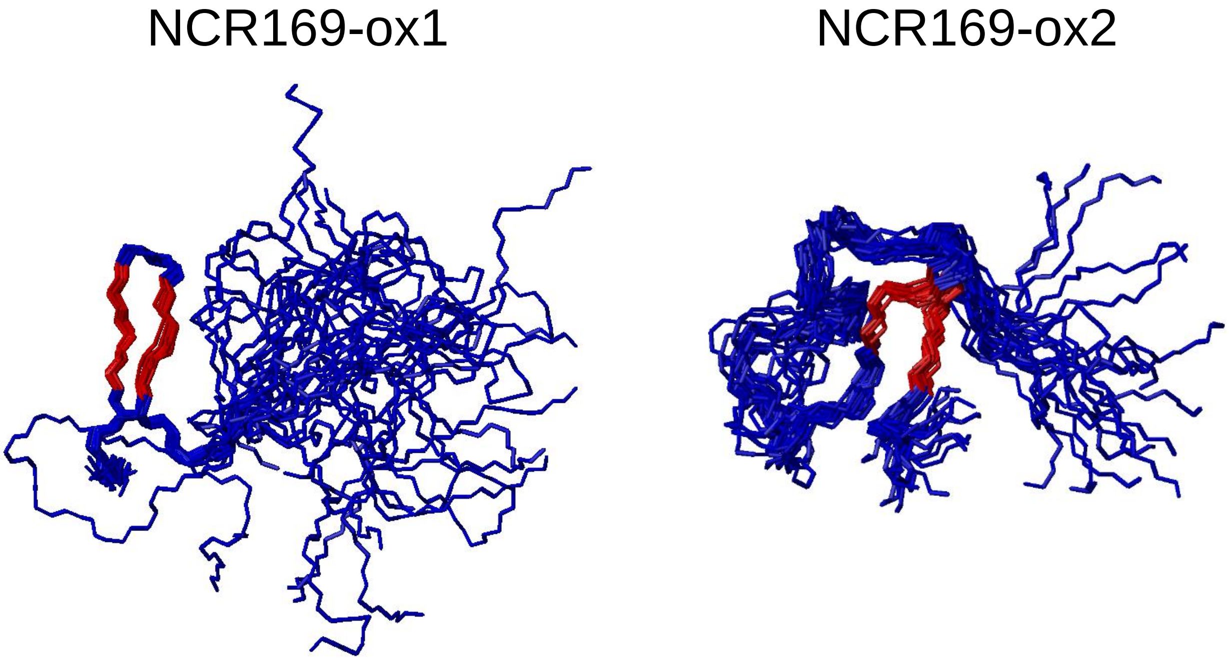

Supplementary Figure S5. 20 NMR structures of NCR169-ox1 (left) and NCR169-ox2 (right). Only their backbones are depicted. The C-terminal β-sheet is shown in red.

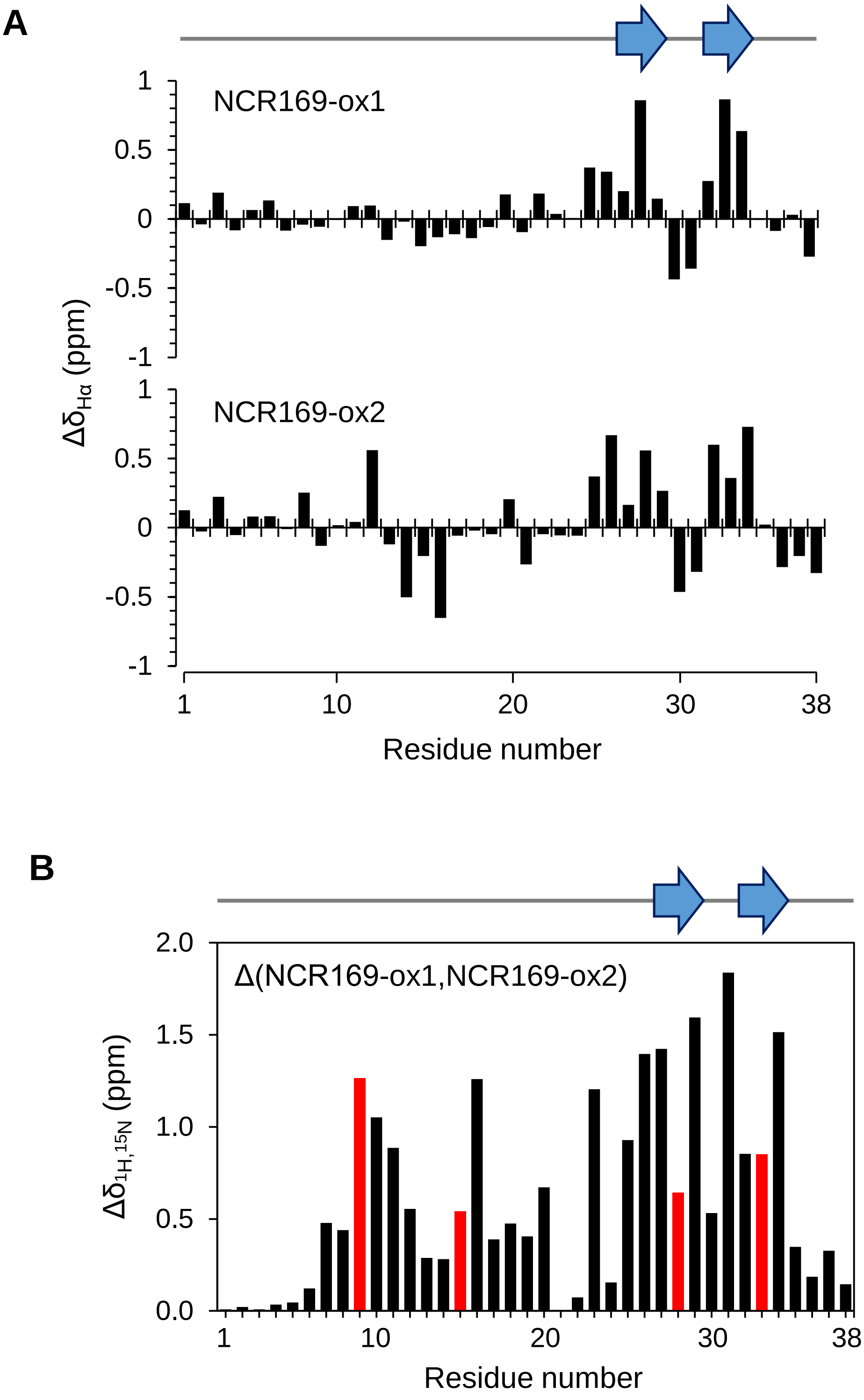

Supplementary Figure S6. (A) The secondary chemical shifts of NCR169-ox1 and -ox2. (B) The chemical shift difference ($\Delta\delta_{^{1}H,^{15}N}$) between NCR169-ox1 and NCR169-ox2. The $\Delta\delta_{^{1}H,^{15}N}$ values were calculated as described in Materials and methods. The bars of cysteine residue are shown in red. Two β-strands (blue arrows) based on the structure calculation are shown above both panels.

Supplementary Table S1. Summary of restraints and statics for the final 20 structures of NCR169-ox1 and -ox2.

Restraints and statics	NCR169-ox1	NCR169-ox2
Restraints		
Total Number of NOEs	362	429
Short range $\|i - j\| \leq 1$	296	294
Medium range $1 < \|i – j\| < 5$	21	40
Long range $5 \leq \|i – j\|$	45	95
Dihedral angle restraints (phi, psi)	7, 7	7, 7
Hydrogen bond restraints	2	2
Structure statistics		
CYANA target function value ($Å^2$)	0.13	0.11
Maximum residual distance constraint violation (Å)	0.22	0.19
Average pairwise RMSD (Å)		
Backbone atoms (residues 24-38)	0.29 ± 0.11	0.71 ± 0.21
Heavy atoms (residues 24-38)	0.86 ± 0.18	1.64 ± 0.24
Ramachandran plot (%)		
Most favored regions	45.1	45.9
Additionally allowed regions	44.1	40.1
Generously allowed regions	8.2	10.6
Disallowed regions	2.5	3.4

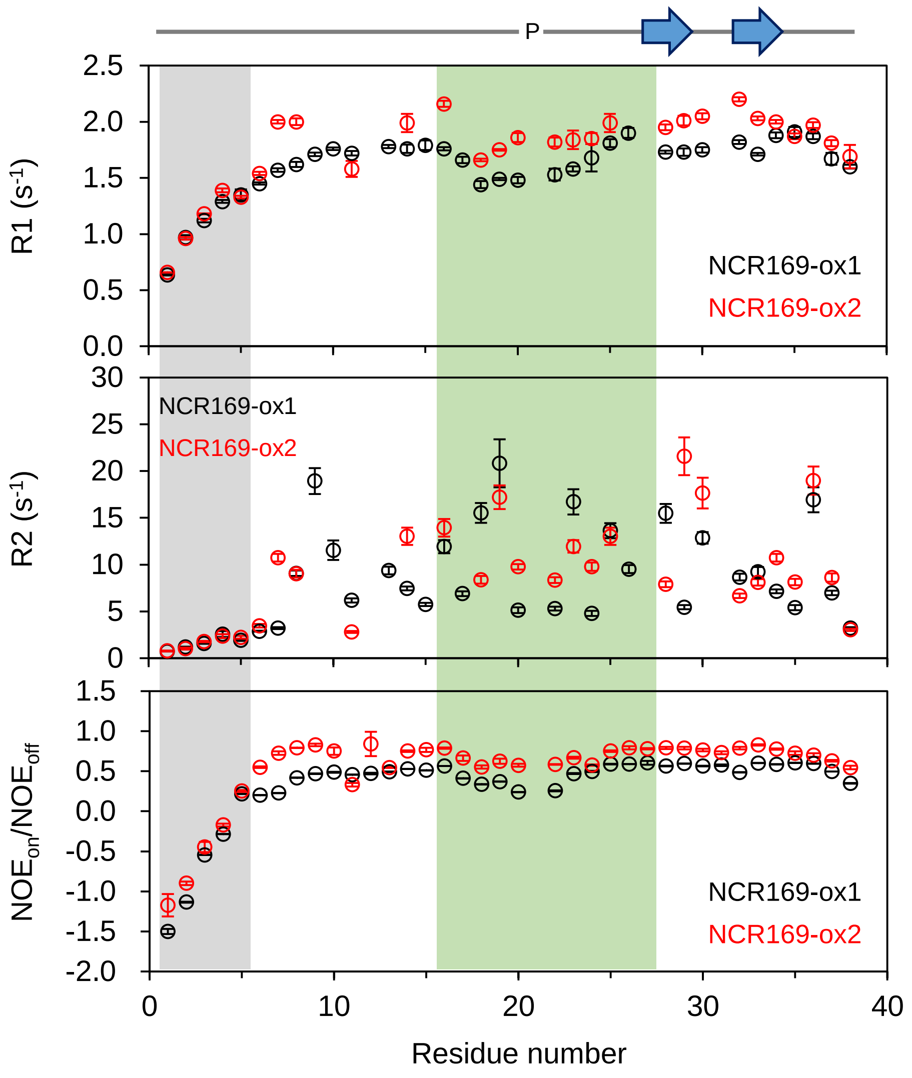

Supplementary Figure S7. The results of NMR relaxation experiments for NCR169-ox1 (black) and NCR169-ox2 (red). The T_1 and T_2 values are shown as R_1 (=1/T_1) and R_2 (=1/T_2), respectively. In R_1 and R_2 plot, error bars represent standard error (SE). In NOE_{on}/NOE_{off} plot obtained from {^{1}H}-^{15}N nuclear Overhauser effect (NOE) experiments, error bars represent standard deviation (SD) (n=2). A proline residue (P) and two β-strands (blue arrows) are shown above the graph. The five N-terminal residues (residues 1 to 5, colored gray) have small values, indicating high flexibility. The central Lys-rich region is colored green.

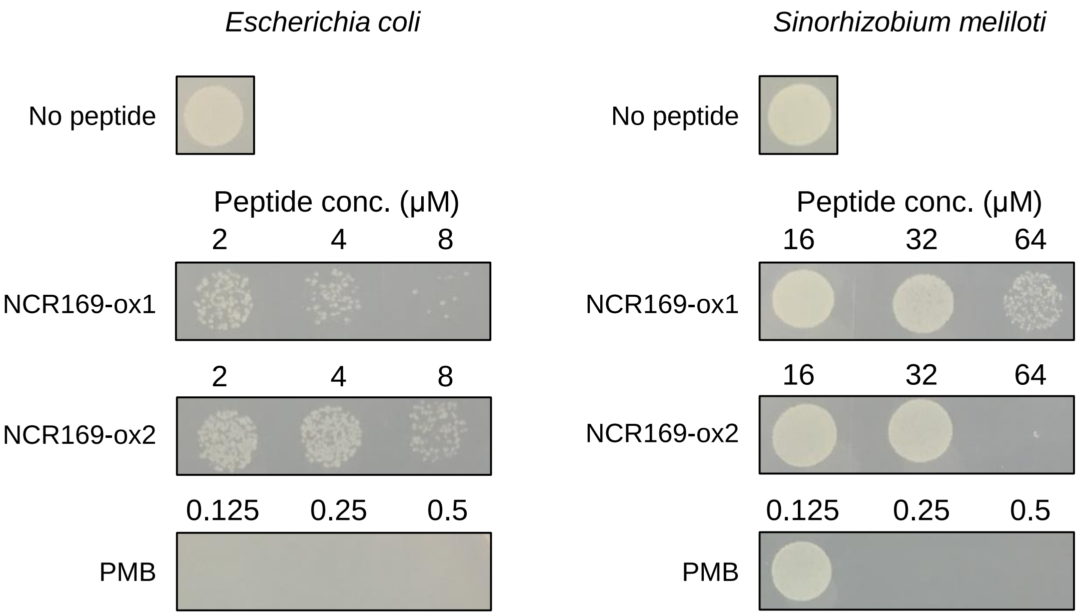

Supplementary Figure S8. Antimicrobial tests using NCR169 peptides (NCR169-ox1 and -ox2) and polymixin B (PMB) against *Escherichia coli* K-12 and *Sinorhizobium meliloti*.

Supplementary Table S2. Antimicrobial activities of NCR169 peptides against *E. coli* K-12 and *S. meliloti*.

Peptide	IC_{50} (µM)*		IC_{100} (µM)	
	E. coli K-12	*S. meliloti*	*E. coli* K-12	*S. meliloti*
PMB	0.018	0.092	0.031	0.25
NCR169-ox1	0.41	6.4	16	128
NCR169-ox2	3.4	9.8	16	128
NCR169N-ox	N/A	N/A	>128	>128
NCR169M	0.083	0.24	0.5	2
NCR169CS-ox	5.6	45	64	>128
NCR169CL-ox	0.14	0.57	0.5	>128

IC_{50}: 50% inhibitory concentration (Calculated using “Dose-response curve” fitting)

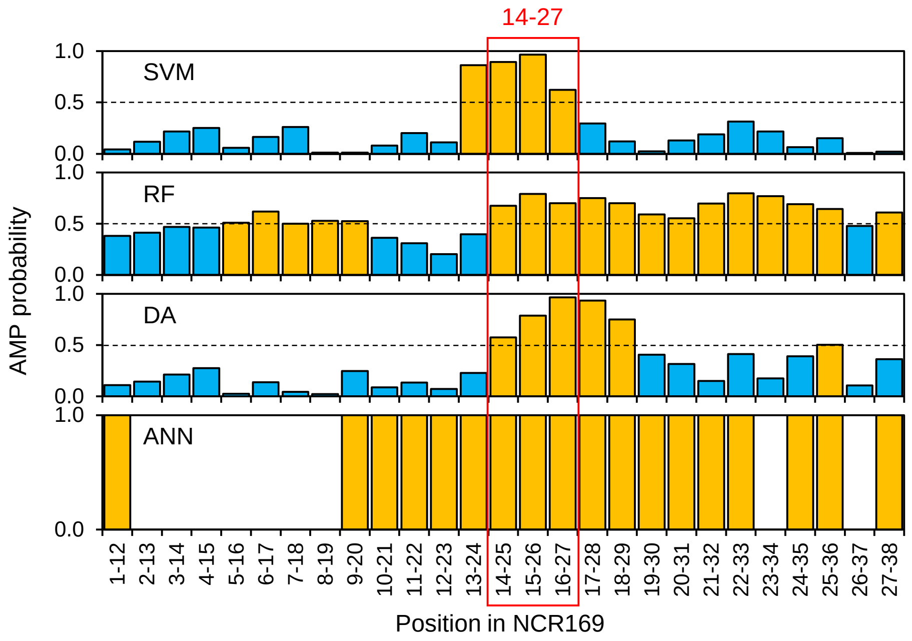

Supplementary Figure S9. Antimicrobial region prediction of NCR169. The prediction was performed using four algorithms, Support Vector Machine (SVM), Random Forest (RF), Artificial Neural Network (ANN), and Discriminant Analysis (DA) on the collection of anti-microbial peptides (CAMP). The antimicrobial peptide (AMP) probability obtained from each algorithm was shown as a bar graph. For the ANN algorithm that provided no AMP probability, AMP and NAMP (non-antimicrobial peptide) were set to 1 and 0, respectively. The orange bar indicates the antimicrobial region. The red box (positions 14 to 27) means the antimicrobial region predicted by all four algorithms.

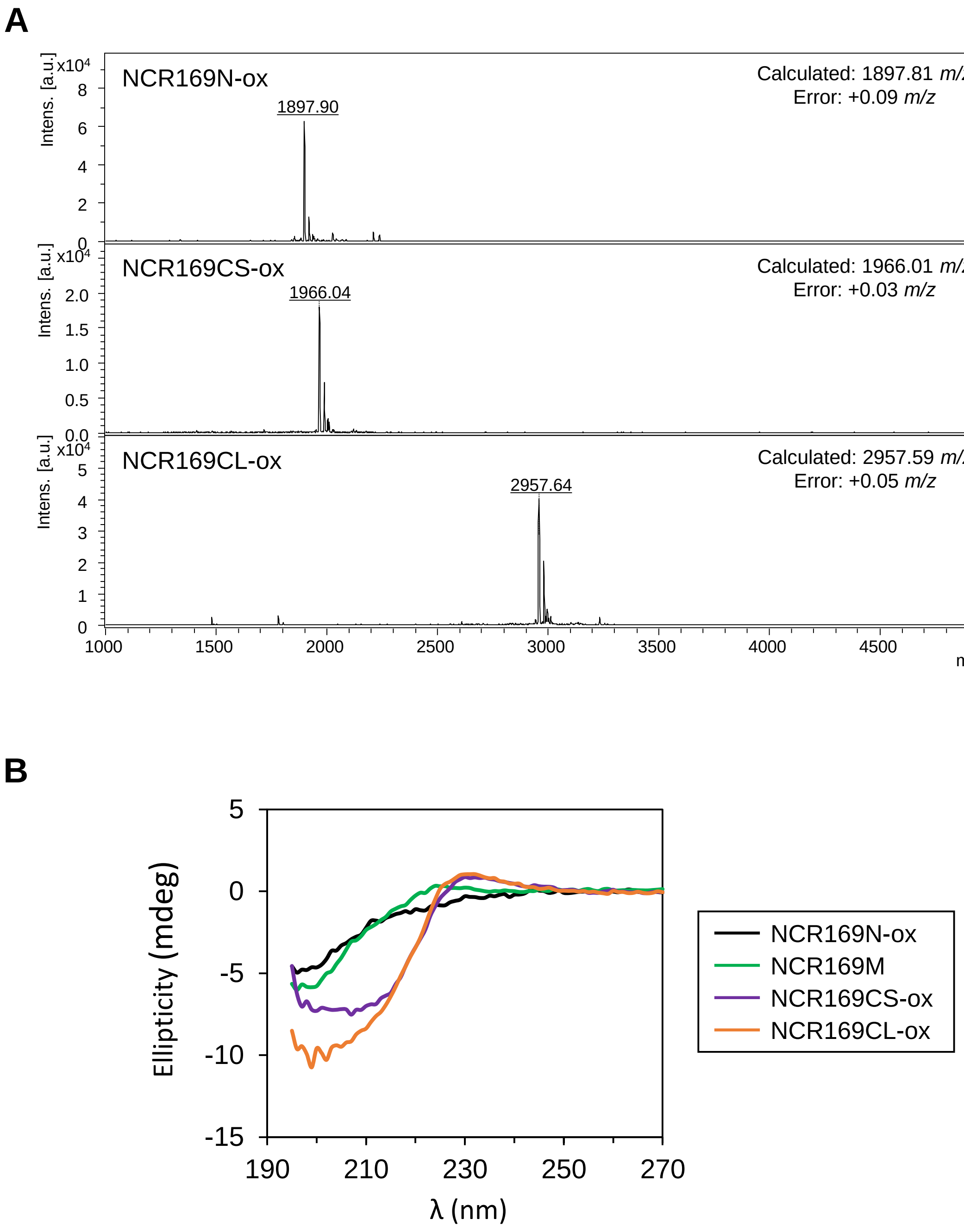

Supplementary Figure S10. Sample preparation of NCR169-derived peptides. (A) MALDI-TOF-MS spectra of NCR169N-ox (upper), NCR169CS-ox (middle), and NCR169CL-ox (lower). (B) Circular dichroism (CD) spectra of NCR169-derived peptides (20 μM). NCR169CS-ox and NCR169CL-ox have the β-sheet indicating signal (218 nm).

A

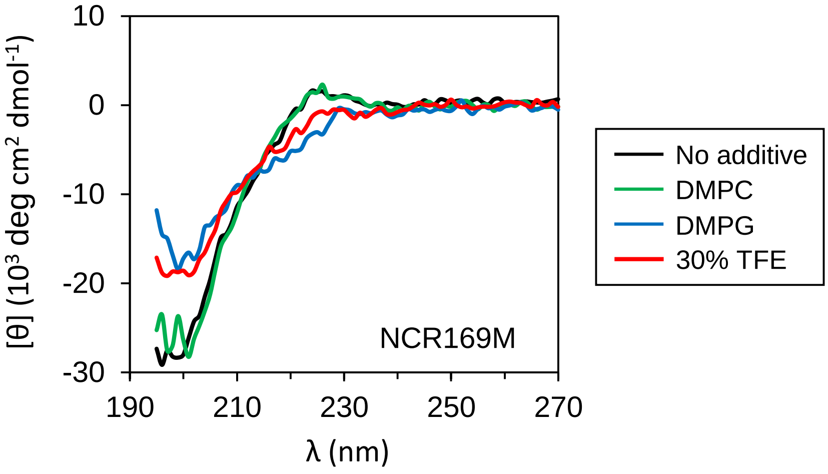

B

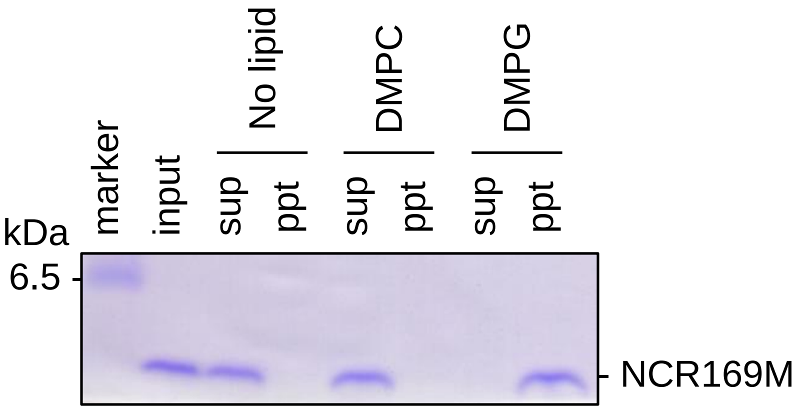

Supplementary Figure S11. NCR169M binds to the bacterial phospholipid. (A) Circular dichroism (CD) spectra of NCR169M (20 μM) in water (No additive), 2 mM dimyristoylphosphatidylcholine (DMPC) liposomes, 2 mM dimyristoylphosphatidylglycerol (DMPG) liposomes or 30% 2,2,2-trifluoroethanol (TFE). (B) The result of liposome binding assay. NCR169M (20 μM) was incubated with buffer (No lipid), 2 mM DMPC liposomes, or 2 mM DMPG liposomes. After incubation at room temperature for 30 min, the mixture was ultra-centrifuged at 200,000 × g. The supernatant and pellet fractions were analyzed by Tricine-SDS-PAGE. The no lipid sample before ultra-centrifugation was used as the input.

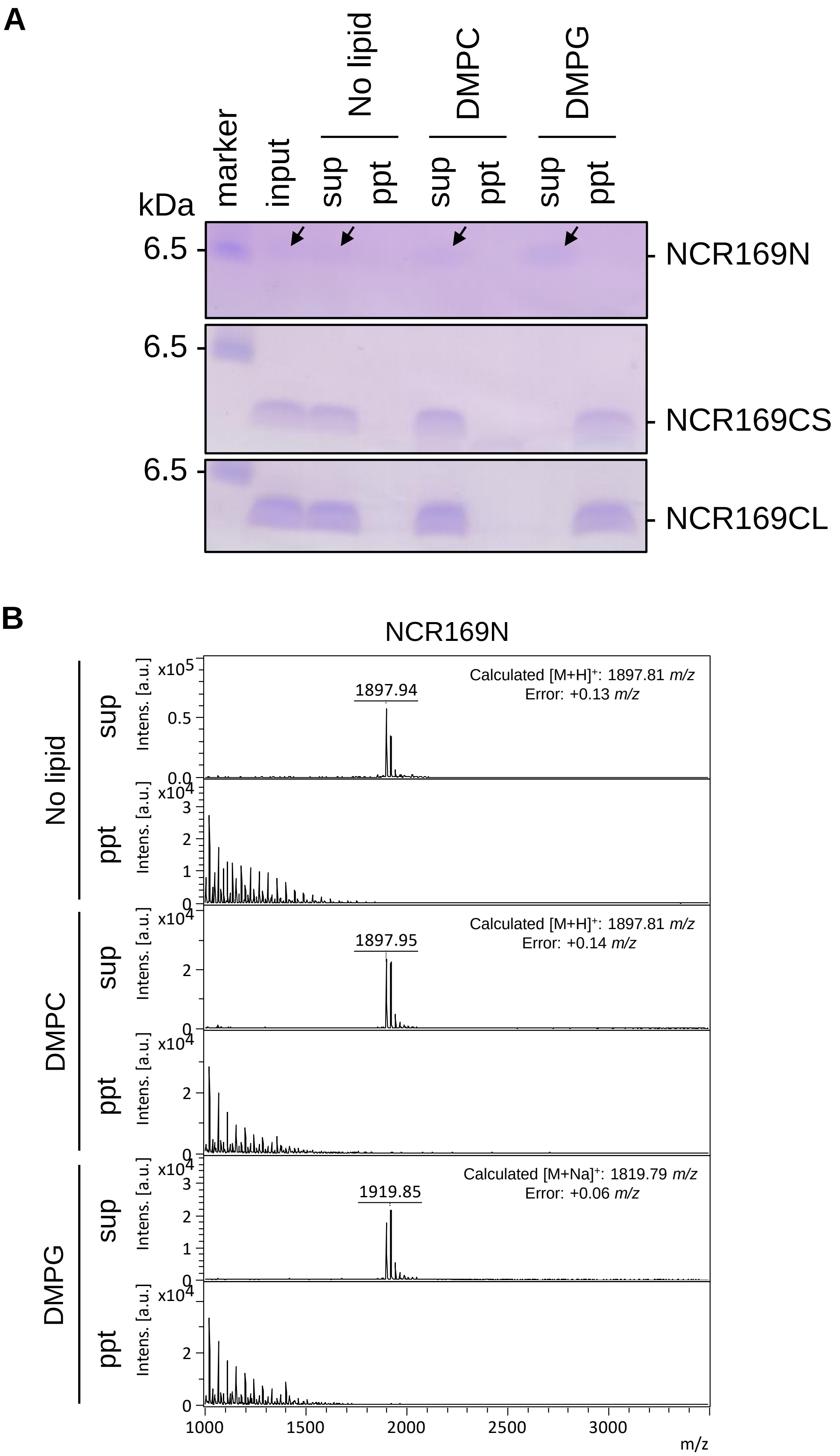

Supplementary Figure S12. The results of liposome binding assays. (A) NCR169-derived peptide (10 μM) was incubated with buffer (No lipid), 1 mM DMPC liposomes, 1 mM DMPG liposomes. After incubation at room temperature for 30 min, the mixture was ultra-centrifuged at 200,000 × g. The supernatant and pellet fractions were analyzed by Tricine-SDS-PAGE. The no lipid sample before ultra-centrifugation was used as the input. Arrows indicate weak bands. (B) MALDI-TOF-MS spectra of NCR169N samples after the liposome binding assay.

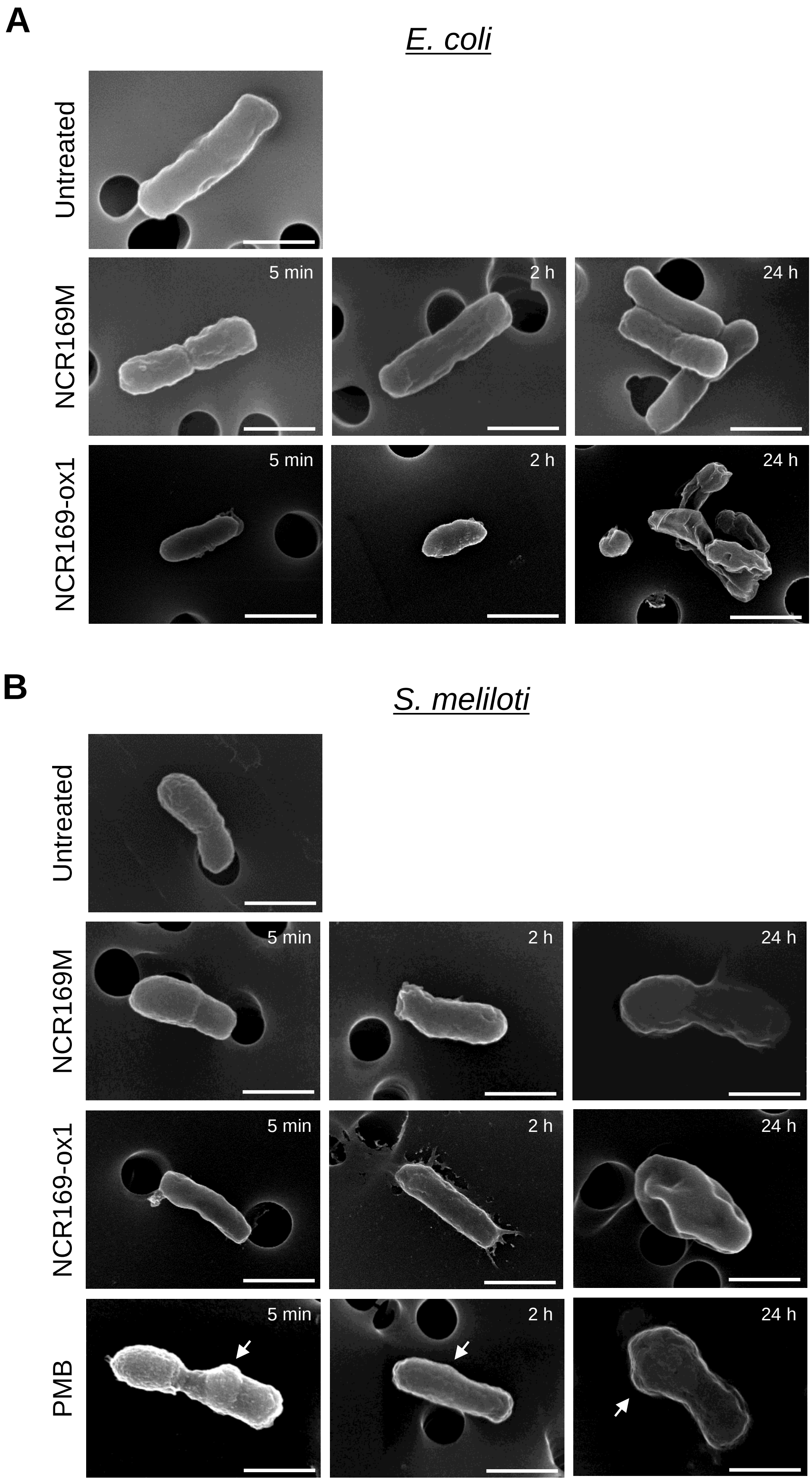

Supplementary Figure S13. Scanning electron microscopy (SEM) images of bacteria cells. (A) SEM images of *E. coli* K-12 untreated or treated with NCR169M or NCR169-ox1. (B) SEM images of *S. meliloti* untreated or treated with NCR169M or NCR169-ox1 or polymixin B (PMB). Arrows indicate cell swelling. All treatments were performed at IC_{100} for 5 min, 2 h, and 24 h. (Scale bars: 1 μm.)
